# Two problematic structures: partial residual electron density in a crown ether and cages with 4 metal centers and 8 disordered anions.

**DOI:** 10.1063/4.0001023

**Published:** 2025-10-27

**Authors:** Veronica Carta

**Affiliations:** UC Riverside, Riverside, CA, USA

## Abstract

I will discuss two problematic structures. I have decided not to publish these structures because I am not satisfied with the refinement, but I think they are worth discussing.

The first structure is a Ni complex with Cs cations, 18-Crown-6, and residual electron density in one of the crown ether’s cavity. The residual electron density could be due to a cation or more likely a water molecule with partial occupancy, but it could not be successfully modeled.

The second structure is a large cage with 4 metal centers and 8 disordered anions. The data quality is not very good, and the anions are highly disordered. Disorder could be modeled, but the resulting structure has poor refinement parameters.

